# Reference genes in aging: what are you referring to?

**DOI:** 10.18632/aging.204710

**Published:** 2023-05-02

**Authors:** Manish Mishra, Susan E. Howlett

**Affiliations:** 1 Department of Pharmacology, Dalhousie University, Halifax, Scotia Nova Scotia 15000, Canada; 2 Department of Medicine (Geriatric Medicine), Dalhousie University, Halifax, Nova Scotia Nova Scotia 15000, Canada

**Keywords:** sex differences, frailty, healthspan, frailty index

Geroscience research aims to understand how aging drives so many chronic diseases and debilitating conditions [1]. Activation of interconnected subcellular and cellular mechanisms, known as “pillars” or “hallmarks” of aging, are thought to lead to adverse remodeling, thereby setting the stage for late-life diseases [1, 2]. The resulting damage at the molecular level scales up to adversely affect cells, tissues and then organs leading to system failure [3]. Individuals who accumulate the most damage experience accelerated aging, also known as frailty [3]. The growing interest in how aging and frailty mechanisms respond to therapeutic interventions has motivated studies of their impact on gene expression in various tissues.

To measure the effects of age on gene expression, quantitative-polymerase chain reaction (qPCR) studies normalize expression levels to one or more reference genes. As they serve as internal controls, it is vital that these genes are stably expressed under the different conditions used in any given experiment [4]. Such reference genes generally are constitutively expressed, being involved in basal cellular functions such as protein synthesis or metabolism [4]. Because commonly used reference genes involve enzymes and proteins that regulate major biological processes, they may be modified by age or overall health status (measured as frailty). It seems likely that they differ between the sexes. Even today however, most studies that have investigated reference gene stability have used only one sex, and in almost all cases they have used males [4]. This is not without scientific interest; aging is expressed differently in men and women. Why should we not expect to see differences in reference gene expression?

**Figure 1:**
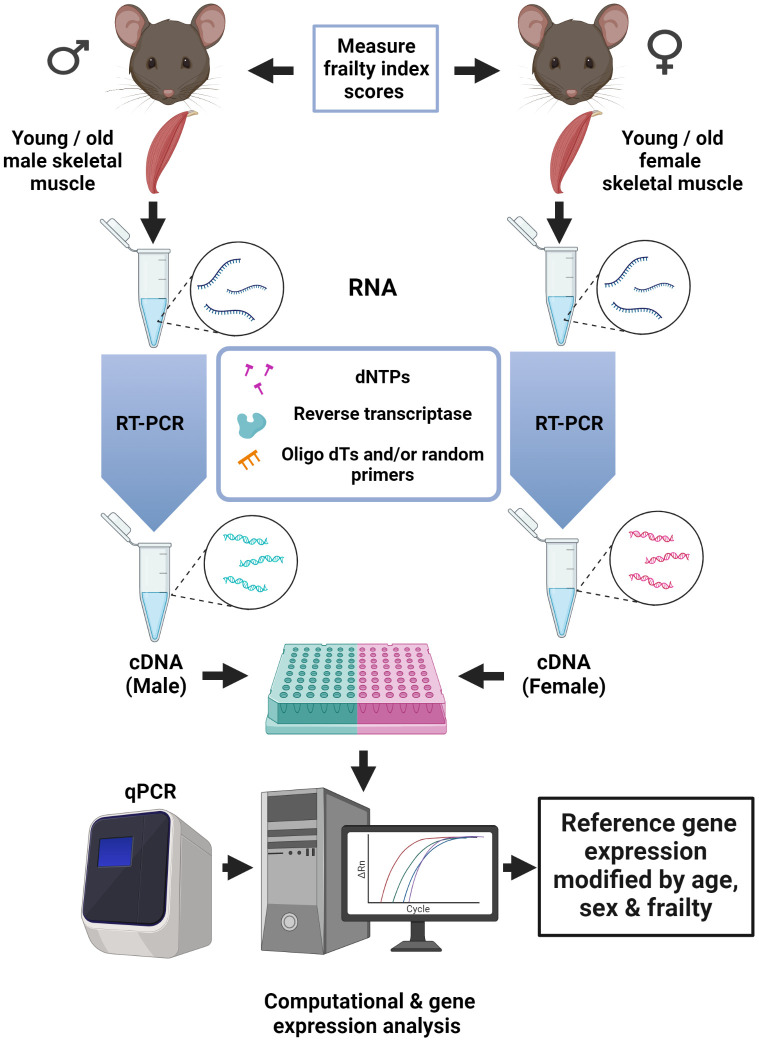
**Age, sex, and overall health affect reference gene expression in skeletal muscle from C57BL/6 mice.** A schematic diagram that illustrates the experimental approach used to compare reference gene stability in skeletal muscle (hamstrings) from young (4 mos) and old (25-26 mos) mice. Eight common reference genes were investigated (Gapdh, Gusb, Rplp0, B2m, Tubb5, Rpl7l1, Hprt, and Rer1). Health was assessed with a validated frailty index tool. Figure created with Biorender.com.

Our recent study, published in Mechanisms of Ageing and Development, explored the impacts of age, sex and frailty on the expression and stability of eight commonly used reference genes (Gapdh, Gusb, Rplp0, B2m, Tubb5, Rpl7l1, Hprt, Rer1) in skeletal muscle samples from C57BL/6 mice [5]. The general experimental approach used in our studies is illustrated in Figure 1. Our analysis of raw quantification cycle (Cq) values showed that only one of the eight reference genes examined (Rplp0; ribosomal protein large P0) was stable in both sexes at both ages. We then used this stable reference gene to normalize our qPCR data and found that age affected the expression of most reference genes examined (Hprt, Gusb, Rer1, Rpl7l1), but only in females. A similar sex-specific pattern was seen when we normalized our data to Gapdh (selected by the standard reference gene selection programs RefFinder, Genorm and NormFinder) or to the Rplp0/Gapdh gene pair. Critically, we measured overall health with a mouse frailty index tool based on an instrument used to assess the degree of frailty in people [3]. We reported the novel finding that frailty modifies reference gene expression. As with age, the impact of frailty was much more pronounced in females than in males [5].

These findings are important. Females have long been underrepresented in both clinical and preclinical studies, leading to calls from bodies such as the National Institutes of Health to address this sex imbalance [6]. Research in this area is increasing [7], but as we see important gaps persist. So too does an invidious piece of biomythology – that in females the estrous cycle invalidates comparisons. This myth has been repeatedly debunked in various ways. For example, group-housed female mice do not even exhibit regular estrous cycles [8]. Here we show that age-related changes in reference gene expression occur primarily in females, so the stability of a reference gene in aging cannot be assumed from data obtained in males. In addition, our unique observation that health status affects reference gene expression, especially in females, is also vitally important. The biological functions of commonly used reference genes like those employed in our study relate to metabolism, immune function and protein synthesis, all systems that are identified as important pillars or hallmarks of aging and frailty [1, 2]. It is perhaps not surprising that these processes can be modified by age, sex and/or health status.

This work underscores the importance of taking age, sex, and the degree of frailty into account when selecting reference genes to normalize mRNA abundance data. It also opens up many areas to be explored. For starters, we used eight common reference genes, but others should be investigated. Our findings apply to fast-twitch muscle from variably aged C57BL/6 mice of both sexes. Whether similar changes would be seen in other tissues and even other types of skeletal muscle is not yet clear. It will be critically important to examine whether reference gene expression is affected by these factors in human samples as well. This could be accomplished in several ways. For example, clinical studies should investigate whether sex differences are present, rather than simply controlling for sex as typically persists still. In addition, relationships between reference gene expression and frailty could be investigated with one of the frailty index tools developed for use in people [3]. If factors such as age, sex and frailty are not considered in experimental studies, we will continue to have no clear idea what we are referring to.
